# Eligibility for randomized trials of treatments specifically for intracerebral hemorrhage: community-based study

**DOI:** 10.1186/1745-6215-14-S1-P47

**Published:** 2013-11-29

**Authors:** Arthur Fonville, Neshika Samarasekera, Yvo Roos, Rustam Al-Shahi Salman

**Affiliations:** 1 Centre for Clinical Brain Sciences, University of Edinburgh, Edinburgh, UK; 2 Academic Medical Center, University of Amsterdam, Amsterdam, The Netherlands

## Background

There are no acute treatments specifically for intracerebral hemorrhage (ICH), but they are being sought in randomized controlled trials. The treatment effect sizes in ongoing and future trials are likely to be small, necessitating large sample sizes.

## Methods

We searched online trial registries for randomized controlled trials investigating an acute treatment for ICH. For the trials whose eligibility criteria could be assessed in a prospective, community-based ICH cohort study (2010-11), we quantified the proportions of patients who were eligible and investigated influences on these proportions.

## Results

We applied the eligibility criteria of 17 trials to 166 adults with ICH, of whom between 0.6% (95% confidence interval [CI] 0.1-3.3) to 40% (95% CI 33-48) were eligible for each trial. Fewer patients were eligible for trials restricted to patients randomized within 12 hours of ICH onset compared to trials with a longer time window (p=0.03). Each additional eligibility criterion reduced the portion of eligible patients by 1.3% (95% CI 0.4-2.2; adjusted R2 = 0.47; p=0.004). At least 66% (95% CI 58-73) of the entire cohort was ineligible for all of the six trials that were ongoing at the time of this study.

## Conclusion

Fewer than half of patients with ICH are eligible for current randomized controlled trials. Future trials could maximize enrollment by extending the time window after ICH onset for recruitment and minimizing the number of eligibility criteria. We are developing an online trial eligibility simulator based on our dataset to assist investigators planning future trials of treatments for ICH.

